# Accelerating skin regeneration and wound healing by controlled ROS from photodynamic treatment

**DOI:** 10.1186/s41232-022-00226-6

**Published:** 2022-10-04

**Authors:** Khatereh Khorsandi, Reza Hosseinzadeh, HomaSadat Esfahani, Kavosh Zandsalimi, Fedora Khatibi Shahidi, Heidi Abrahamse

**Affiliations:** 1 Department of Photodynamic, Medical Laser Research Center, Yara Institute, ACECR, Tehran, Iran; 2 Department of Biochemistry and Molecular Medicine, School of Medicine and Health Sciences, The George Washington University, Washington, DC, 20037 USA; 3 Academic center for education, culture and research, Urmia, Iran; 4 Laser Research Centre, Faculty of Health Sciences, University of Johannesburg, P.O. Box 17011, Doornfontein, Johannesburg, 2028 South Africa

**Keywords:** ROS, Oxidative stress, Photodynamic therapy, Skin regeneration, Wound healing

## Abstract

Cellular metabolisms produce reactive oxygen species (ROS) which are essential for cellular signaling pathways and physiological functions. Nevertheless, ROS act as “double-edged swords” that have an unstable redox balance between ROS production and removal. A little raise of ROS results in cell proliferation enhancement, survival, and soft immune responses, while a high level of ROS could lead to cellular damage consequently protein, nucleic acid, and lipid damages and finally cell death. ROS play an important role in various pathological circumstances. On the contrary, ROS can show selective toxicity which is used against cancer cells and pathogens. Photodynamic therapy (PDT) is based on three important components including a photosensitizer (PS), oxygen, and light. Upon excitation of the PS at a specific wavelength, the PDT process begins which leads to ROS generation. ROS produced during PDT could induce two different pathways. If PDT produces control and low ROS, it can lead to cell proliferation and differentiation. However, excess production of ROS by PDT causes cellular photo damage which is the main mechanism used in cancer treatment. This review summarizes the functions of ROS in living systems and describes role of PDT in production of controllable ROS and finally a special focus on current ROS-generating therapeutic protocols for regeneration and wound healing.

## Introduction

Photodynamic therapy (PDT) has been applied for cancer treatment, infections, and inflammatory situations, such as acne, rosacea, and genital warts [1]. PDT defines as a mixture of a chemical compound, known as a photosensitizer (PS), and light at specific wavelengths which led to a series of photochemical reaction that subsequently leads to cellular damage [2]. ROS are a major product of PDT, which plays a key role in intracellular signal transduction regulation in vivo. Whereas the specific mechanisms of regulation have not yet been explained, ROS can target different signaling pathways in the cell [3].

It has been demonstrated that PDT remodels extracellular matrix by modulation of collagen synthesis or photosensitization of collagens [4]. In addition, PDT reduces the migration and invasion ability of cancerous cells by downregulation of several matrix metalloproteinases (MMPs) [5]. ROS also induce endoplasmic reticulum (ER) stress and enhance secretion of damage-associated molecular patterns (DAMPs) which trigger immunogenic apoptosis. PDT-induced apoptosis is known as a safe and efficient treatment modality in malignant carcinomas [6].

PDT dose is a key element in identifying concentration of ROS during photochemical reactions [7]. In contrast to high levels of ROS which lead to cellular toxicities, low-dose PDT can induce cellular proliferation and differentiation [8], consequently provoking the differentiation of pluripotent stem cells, including mesenchymal stem cells [8] and neural stem cells [9]. It has been shown how exogenous ROS can affect stem cells in vitro [10]. It has been demonstrated that in situ ROS generation in murine skin triggered hair follicle stem cell proliferation, inducing hair growth and healing [9]. However, the specific effects of ROS generated by PDT on skin regeneration and wound healing are unknown. It is noted that lower doses of PDT may involve skin regeneration stimulation compared to higher doses which has been used in killing cancer cells. Recently, we investigated that low-dose PDT enhanced wound healing, without significant cytotoxicity in vitro [11].

This review will talk about the physiological function of ROS with an emphasis on its role in PDT for skin rejuvenation and wound healing.

## ROS definition, generation, and its physiological roles

Oxygen-derived molecules are generated by reductive-oxidation reactions (redox oxidation) or by electron excitation to form a group of molecules called ROS. ROS is a term and is not chemically accurate. However, because of complications in distinguishing between singular ROS varieties, a familiar convention in redox biology has been known to use “ROS” as a superset [2].

ROS can be described as oxygen-containing reactive species. This collective term includes hydrogen peroxide (H_2_O_2_), superoxide (O_2_^▪−^), singlet oxygen (^1^O_2_), hydroxyl radical (OH^▪^), alkoxyl radical (LO^▪^), lipid hydroperoxide (LOOH), peroxyl radical (LOO^▪^), hypochlorous acid (HOCl), and ozone (O_3_), and peroxynitrite (ONOO^−^), among others. In addition to ROS, there are other terms used in articles to describe reactive oxygen species, such as reactive oxygen intermediate (ROI), reactive oxygen metabolite (ROM), and oxygen radicals. ROS is the most well-known among these diverse terms [12].

ROS produced from various sources and several intracellular mechanisms regulate the generation of ROS to maintain their physiological concentration which will be discussed later.

There are three main sites to produce oxidants within the cells: mitochondria, peroxisomes, and endoplasmic reticulum (ER). Each of these sections is equipped with its own antioxidant system that prevents cell damage and protects intracellular functions. Free radicals or ROS and especially singlet oxygen are produced in the mitochondrial respiration chain (electron transfer cycle). The level of active electron compounds (ROS) within cells is maintained due to the balance between the production of oxidants and the concentration of antioxidants in cells. Under normal circumstances, mitochondrial antioxidants including super oxide dismutase (SOD) and glutathione (GSH) are abundant to offset these active species and defend mitochondrial integrity. Impaired activity of a small number of mitochondria can lead to suppressed ATP production and unregulated ROS release. In several sections along the respiratory chain, electrons derived from NADH or FADH can react directly with oxygen or other electron receptors to produce ROS [13]. To the best of our knowledge, the ER has a critical role in protein folding and Ca^2+^ homeostasis, and dysfunction of the ER leads to disruption of the protein folding process which cause the ER stress. Unrestrained ER stress leads to abnormal regulation of Ca^2+^, induction and release of ROS, and activation of apoptosis pathways and autophagy. Peroxisomal role is also related to the function of both cellular components, namely mitochondria and ER, and therefore their dysfunction causes the production and release of H_2_O_2_ in the cellular environment. It should be noted that various factors such as drugs, environmental toxins, and aging play a role in cell organelle damages and causes the ROS production resulting in cardiovascular disease, neurological diseases, various cancers, and chronic wounds [14].

In human cells, 41 enzymes which producing H_2_O_2_ and O_2_ have been identified, and this number has reached more than 50 with the addition of enzymes producing other active species of oxygen such as hypochlorous acid and lipid hydroperoxides or nitric oxide (NO) [3]. NADPH oxidases (NOXs) and electron transfer chains (ETCs) are the main sources of endogenous enzymatic production of O_2_^▪−^ and H_2_O_2_ [15, 16]. Specific redox-active endosomes associated with NOXs are activated in response to extracellular stimuli such as nutrients, growth factors, and cytokines, and aid compartmentalization of H_2_O_2_ for localizing redox-mediated regulation (microdomains) or cell signaling from cell surface receptors [17]. Complexes I and II in the electron transfer chain in the mitochondria release O_2_^▪−^/ H_2_O_2_ to the mitochondrial matrix while complex III is discharged to the cristae lumen and intermembrane space [18]. The functional significance of this topological diversity is revealed through variable redox-modified proteins depending on their origin. The ER and peroxisomes are responsible for the local production of H_2_O_2_ from O_2_^·^ through various SODs (SOD1-SOD3). In addition to the biology of O_2_^▪−^ / H_2_O_2_, a significant zone of ROS research is related to lipid-derived ROS in which polyunsaturated fatty acids are oxidized to produce lipid hydroperoxides and related radicals, peroxyl and alkoxy, and have a major impact on redox signaling [19], especially in immune signaling [20]. For example, reactive oxidants are synthesized by lipoxygenases and prostaglandin synthases act as intermediates to control inflammatory responses [21].

The production of predominant intracellular oxidant generators remains a major question. The latest estimate of stopped myoblasts shows that 40% of cellular H_2_O_2_ production is balanced by NOXs and approximately 45% by ETC, with production levels from other enzymatic origins [22]. Therefore, participation of NOXs and the ETC is equivalent. Cell connection and cellular metabolic position determine the diversity of ROS sources and their specific distribution. Also, intracellular, oxidants are produced as an effect of the combined environmental exposure described the “exposome,” which involves molecular factors such as toxicants, nutrients, drugs, and pollutants as well as physical stressors (UV, X-ray, and other ionizing radiation) and psychological stressors (lifestyle). As mentioned, endogenous sources of ROS production are mitochondrial electron transfer chain and NAD(P)H oxidases. Exogenous sources of ROS production include xenobiotics produced during oxidation cycles such as redox, air pollution, and radiation. The amount of ROS produced in the biological system is measured through the production of ROS, as well as the activation of the cellular antioxidant system. GSH wasted by electrophiles leads to the production of secondary oxidative stress. Metabolization of environmental chemical compounds such as drugs causes the formation of electrophilic metabolites.

In addition to ROS, other by-products such as RONS (active species of nitrogen) are produced during redox processes in the cell [23]. These compounds include nitrogen and oxygen radicals and non-radical active compounds [24]. Free radicals are generated from exogenous ROS and RONS related to air pollution, drugs, alcohol, tobacco, heavy metals or intermediates, food products, water, and radiation. The main origins of ROS within a cell are enzymes. Myeloperoxidase (MPO), lipoxygenase, angiotensin II, and nicotinamide adenine dinucleotide phosphate (NADPH) oxidase are the endogenous RONS sources [14].

Apart from these origins, other important sources of endogenous oxidants involve nitric oxide synthase, cytochrome p450, monoamine oxidase, various oxidoreductases such as mitochondrial respiratory chain (RC), xanthine oxidase, and enzymes responsible for infection and inflammatory responses to stimulate xenobiotic for instance NADPH oxidases [25]. Studies showed that by increasing age and pathophysiological conditions, the production of oxidants from these sources enhances [26, 27]. ROS derivatives or oxidative compounds can easily convert to radicals [28]. ATP production by mitochondria leads to the production of free radicals. Other aerobic mechanisms such as cellular respiration, bacterial infections that involve the activation of phagocytes, and physical respiratory activity also lead to the production of free radicals [29]. Free radicals were first recognized in biological systems in the 1950s and it was assumed that they are engaged in aging and several other pathological conditions [30]. By binding specific molecules to oxygen, free radicals are produced with one or more pairs of unpaired electrons on their outer surface. These active free radicals act as oxidants or reductants depending on whether they receive or lose single electrons [14].

In terms of electric charge, free radicals are neutral, negative, or positive. Diatomic oxygen O_2_ is an example of a radical having two unpaired electrons. While both electrons have the same spin quantum number, the position of each electron is in the different π* anti-bonding orbital with weak bonding to non-radical molecules with parallel spins. Upon receiving input energy that can reverse the one of the unpaired electrons spins, O_2_ can be turned into noticeably more reactive singlet oxygen O_2_. Both electrons can form the same electron pair in the π * orbital, or they can still be in two different orbitals. Bonding an electron to some oxygen at the same time can break the spin limitation. Natural aerobic respiration as well as stressful situations produces non-radical compounds similar to H_2_O_2_ in the body [28]. Several intracellular activities are performed by ROS, in fact, a balance between oxidants and antioxidants is important for growth, adaptation, regulation, and biological role. ROS-related activities include gene transcription, immune response, cell survival and death, differentiation, inflammation, and cell signaling transduction [31]. Although ROS damages DNA, proteins, and lipids, they play vital roles in the body’s physiology, too. For example, ROS production by phagocytes, as an innate immune component, can kill pathogen-invading microorganisms. ROS is also important as secondary messengers in redox cell pathways. Interactions of antioxidant compounds with these physiological functions of ROS interfere with biological systems. Therefore, overexpression of Nrf2, the major regulator of antioxidant genes, increases tumorigenesis. Drugs also induce ROS production, for example, the metabolism of several anticancer drugs lead to the formation of ROS, which leads to the death of cancer cells. Lately, ROS delivery to cancer cells has been proposed as a tool for cancer treatment [32, 33].

## ROS and development of skin diseases

ROS has major function in various processes in the skin including aging, inflammation, regeneration, and wound healing. Besides, the activity of transcription factors, phosphatases, kinases, and cysteine-rich redox-sensitive proteins can be altered in the presence of ROS. Thus, oxidative stress can significantly affect several physiological processes [34].

The skin naturally has defense mechanisms against redox-active chemicals, UV, and ionizing radiation, which induce excessive production of ROS. Additionally, endogenous antioxidants are devoted to protecting tissues against destructive effects of ROS. However, long-lasting presence and accumulation of free radicals in the tissues restricts the effectiveness of the defense mechanisms and triggers uneven cellular responses associated with skin disorders, photosensitivity, and malignancies [35].

### Role of ROS in pathogenesis of vitiligo

Vitiligo is an acquired pigmentation disorder with 0.5 to 2% global incidence rate [36]. The main characteristic of vitiligo is progressive and continues dyspigmentation of skin which results in development of depigmented patches over the body. The etiology of vitiligo is comprised of a complex interaction of chemical, biological, and environmental factors.

Vitiligo is an autoimmune dermal disease. Numerous genetic and environmental factors contribute to the development of vitiligo, with a relative proportion of 80 to 20%, respectively. These factors include family history, stress, sunlight exposure, skin infections, injuries, and malignancies. The role of melatonin receptor dysfunction and melanocyte migration disorders in pathogenesis of vitiligo has also been recognized. In addition, the association of neural anomalies, endocrine diseases, and some drugs with vitiligo has been demonstrated. These factors act independently or in combination in the susceptibility to vitiligo [37, 38]. On the cellular level, CD8+ T-cells are responsible for the initiation and development of vitiligo. Perilesional skin explants of vitiligo patches contain CD8+ T-cells that kill targeted melanocytes and form depigmented lesions. The recruitment of CD8+ T-cells in active lesions is mediated by intralesional interferon-γ (IFN-γ). In skin and serum, IFN-γ increases CXCL9 and CXCL10 chemoattractant levels in which their function is to attract pathogenic T-cells [39, 40]. On a genetic level, many genes associated with vitiligo have been recognized in genome-wide studies including *MHC classes I and II*, *CD44*, *CD80*, *PTPN22*, *UBASH3A*, *RERE*, *CTLA4*, *SERPINB9*, *IKZF4*, *TYR*, *OCA2*, *MC1R*, *BCL2L12*, *ASIP*, *SH2B3*, *GZMB*, *CASP7*, *FASLG*, *BCL2L11*, *NEK6*, and *BAD*. These genes are mainly associated with immune, apoptotic, and melanocyte regulators [41]. It has been shown that oxidative stress causes structural and functional damages in several peptides and proteins. High levels of H_2_O_2_ oxidate methionine residues of tyrosinase and impair the activity of this melanogenic enzyme. In addition, in patients with vitiligo, direct links have been found between oxidative stress and dysfunction of tyrosine-related protein 1 (TRP1) [42].

Recently, the role of ROS in the onset and progression of vitiligo has been shown in several studies. Besides, excessive levels of ROS have been found in active vitiligo lesions suggesting that high concentrations of ROS induce melanocyte destruction. In vitro and in vivo investigations revealed the relation between ROS levels and vulnerability of melanocytes in vitiligo patients. ROS-induced apoptosis of keratinocytes results in loss of melanocyte attachments at the boundaries of vitiligo patches. ROS also promote the overexpression of p53 and its target genes in melanocytes which induce the release of insulin-like growth factor-binding protein 3 and 7 (IGFBP3 and IGFBP7), matrix metalloproteinase-3 (MMP3), interlukin-6 (IL-6), and prostaglandin-endoperoxide synthase 2 (PTGS2). These factors are known as the characteristics of senescence-associated secretory phenotype (SASP). Other consequences of the p53 overexpression include autophagic cell death, ATP release, and the commencement of degenerative processes. The release of SASP factors and ATP lead to the activation of dendritic cells and disturb the balance between CD8^+^ and Treg cells [43, 44]. These findings collectively suggest the association between ROS generation and immune changes responsible for skin depigmentation in vitiligo.

### Role of ROS in pathogenesis of psoriasis

Psoriasis is a chronic autoimmune disease typified by recurrent inflammation and scaling of skin. The estimated prevalence rate of psoriasis is about 2% of global population [36].

Various overexpressed proinflammatory cytokines, such as interleukins, IFN-γ) and tumor necrosis factors (TNFs), are found in psoriatic lesions, which confirm the role of ROS in the pathogenesis of psoriasis. That is why therapeutic approaches based on antioxidants are effective in the treatment of psoriasis [35, 45].

The nuclear factor-κB (NF-κB), MAPK/AP1, and JAK-STAT participate in the pathogenesis of psoriasis by enhancing the expression of proinflammatory chemokines and cytokines. Meanwhile, ROS modulates these transduction pathways and promote psoriasis development. ROS also activates the MAPK/AP1-signaling axis and participate in activation of ASK1, RAS, MEKK1, and MLK3 receptors. Furthermore, they adapt the expression of the protein kinase Cζ (PKCζ) which is involved in the overexpression of CD1d, a molecule with potential role in keratinocyte-NK-T cell interactions in psoriatic lacerations [46].

These findings suggest that ROS have different roles in the initiation and progression of psoriasis. Therefore, antioxidants could be used effectively for the treatment of psoriasis.

### ROS and skin wound healing

Wound healing is a multifaceted physiological process in which several factors participate as mediators or regulators. The roles of different classes of hormones, growth factors, and cytokines in this multi-step process have been well demonstrated. In addition to these factors, ROS has a key function in harmonization of the wound healing process. Previous studies have revealed that ROS play a critical role in wound healing by mediating intracellular signaling and defending against attacking pathogens. In addition, ROS-mediated activation of transcription factors induces the release of growth factors, which trigger the autocrine/paracrine signaling pathways of wound healing [47].

ROS provides several wound protection mechanisms by decreasing blood flow and activating cellular signals responsible for thrombus formation. They also attract local neutrophils to the wound bed to guarantee bacterial protection. In addition, ROS released from phagocytosis impede bacterial growth. ROS-mediated signals induce the migration of monocytes to the wound site to protect against invading pathogens. Release of ROS at wound edges promotes fibroblast and endothelial cell division and enhances new extracellular matrix (ECM) formation [48].

A well-known example of ROS is H_2_O_2_, which is found in low concentrations at early stages of wound healing. The level of H_2_O_2_ at wound beds increases by the onset of inflammation stage. As the remodeling stage initiates, H_2_O_2_ limits to the wound edges and its concentration decreases [35]. Studies have demonstrated that low levels of H_2_O_2_ are required during wound healing. H_2_O_2_ catalyzes lipid peroxidation and thus increases the level of 4-hydroxy-2-nonenal (4-HNE) which is a critical mediator for repair process. Moreover, there are evidence that H_2_O_2_ promotes neoangiogenesis in regenerating wounds [49].

On the other hand, high concentrations of ROS cause imbalance in oxidant-antioxidant systems and induce oxidative stress. DNA mutations and aberrations and damages of cellular structures are the main consequences of oxidative stress resulting from the extreme accumulation of ROS within cells [35].

### ROS and therapeutic strategies for wound healing

ROS are increasingly used in different treatment modalities to promote wound healing. ROS intermediates can convert into bioavailable O_2_ in the form of hydrogen peroxide (H_2_O_2_), benzoyl peroxide, and tetrachlorodecaoxide. Results from in vitro studies confirm the effectiveness of topical products containing these compounds in enhancement of wound healing process [47, 50].

H_2_O_2_-containing creams tested on ischemic ulcers of Guinea pigs increased blood flow to the wound bed and promoted angiogenesis. H_2_O_2_ added to phosphate-buffered saline (PBS) has also been examined on excisional wounds of mice. The product was tested at two different concentrations to verify the impact of different oxidative stress levels on therapeutic effects. Results showed that high concentrations of H_2_O_2_ (166 mM) deferred wound closure and made no angiogenesis improvements. However, the suspended response was not related to oxidative damage. In contrast, low levels of H_2_O_2_ (10 mM) improved angiogenesis but also did not have significant effects on wound closure. Further investigations revealed that high concentrations of H_2_O_2_ generate a stronger signal to recruit neutrophils at the wound bed. The study showed that H_2_O_2_ may improve the wound healing responses by promoting angiogenesis. However, it remains uncertain whether levels of H_2_O_2_ are able to induce cell-based reparative responses [51].

Glucose oxidase (GO) is another therapeutic choice with the ability to generate ROS. GO-incorporated dressings have been tested on rats with full-thickness diabetic ulcers. After 3–7 days, ROS levels generated by wound fibroblasts were increased. GO also induced initial increase in SOD, GSH, and NO levels. These antioxidants were associated with enhanced neocollagenesis, wound closure, and keratinocyte differentiation [52].

Galvanic zinc–copper microparticles are another therapeutic platform which are able to increase migration of dermal fibroblasts, enhance keratinocyte ROS production, and decrease the secretion of pro-inflammatory cytokines. Using galvanic zinc–copper particles on synthetic epidermis covered by skin fibroblasts showed the modulatory effect of ROS on fibroblast migration [53, 54]. Another category of products used to enhance the wound healing process and limit infections at wound beds are honey-based dressings. Honey is known as a natural source for several antioxidants such as bee defensin-1, methylglyoxal, and glucose oxidase. It also used to increase antimicrobial and regenerative process [55].

Recently, growth factors and recombinant proteins such as platelet-derived growth factor (PDGF) and galectin-1 have been used as efficient therapeutics to promote wound healing through ROS modulation. Galectin-1 is known as a major player in myofibroblast signaling and function.

Injection of recombinant galectin-1 in mice speeds up wound healing process by increasing ROS levels through NADPH oxidase-4 [56]. PDGF also accelerates wound healing by promoting angiogenesis and inducing macrophage, neutrophil, and fibroblast migration [57].

In addition to the abovementioned therapies, several non-invasive physical methods have been evolved to promote wound healing via ROS modulation. The most common methods include hyperbaric O_2_ therapy, laser treatment, and photodynamic therapy.

## Photodynamic therapy

### Photodynamic reaction

**Fig. 1:**
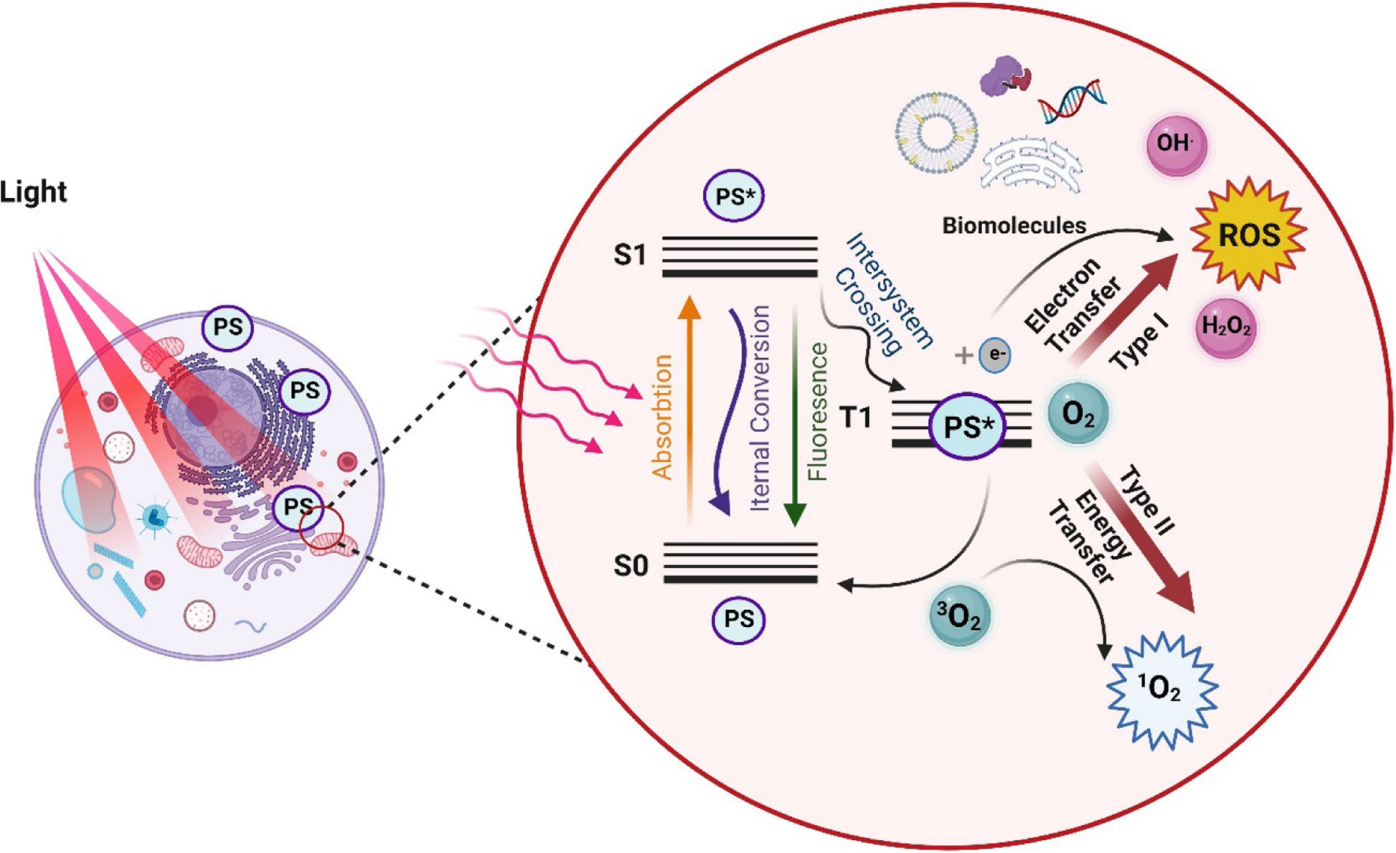
Simplified Jablonski diagram illustrating the formation of singlet oxygen and other reactive species by transferring energy between states and from PS in excited states to molecular oxygen. Type I reaction defines by transferring an electron to biomolecules or directly to oxygen to produce ROS. A type II reaction involves energy transfer from the excited PS to molecular oxygen and producing singlet oxygen

Photodynamic therapy (PDT) was discovered in the early twentieth century when it was discovered that light exposure can kill microorganisms incubated with acridine dyes [52]. Soon after, PDT was widely used therapeutically for oncologic skin with eosin and visible light [53]. Hematoporphyrin products isolated from porcine blood were introduced as the first-generation photosensitizer in human practice [54]. Later, PDT was demonstrated as a very effective clinical treatment especially in specific skin diseases like acne, viral warts, and skin cancers. PDT can be considered as a promising and innovative method for the healing of skin wounds [55, 56]. Photosensitizers are molecules that absorb light (hν) and transfer the energy from the incident light into another nearby molecule. According to the typical photochemical and photophysical signaling of the Jablonski chart, in presence of a PS and light photoactivated to the right excited state of the molecule and then generated reactive species, may stimulate the boosts of ATP generation [57, 58]. The PS by absorbing photons in the ground state (S0) excites to the singlet state (S1) with higher energy. The S1 molecule is able to go back to its S0 state by fluorescence emission or transfer to the triplet excited state (T1) via the intersystem crossing, then produce free radical species by Type I reactions or transferring energy (Type II reactions) to molecular oxygen in the triplet state to the singlet state. Via the known manner that lasts longer than fluorescence, the T1 photosensitizer molecule can also reverse to the S0 stage [59]. All reactions are summarized in Fig. 1.

A type I reaction occurs while a PS in a triplet excited state reacts with an organic element to generate radicals like superoxide anion, hydrogen peroxide, and hydroxyl radicals. Otherwise, in type II reactions, a PS through energy transfers to molecular oxygen directly produces the singlet oxygen that is highly reactive and cytotoxic [60]. The increase in ROS level causes oxidative stress, which results in significant damage to cellular components like DNA, proteins, and lipids [61]. It is important for organisms to be able to reverse the stress and remove or repair damaged elements. Numerous stress reaction mechanisms are quickly activated following oxidative offenses such as the activation of enzymatic and non-enzymatic autoxidizing elements [62, 63]. Nevertheless, ROS attributed to change in diverse signaling pathways and can cause different effects including cell proliferation.

### Photosensitizers

Photosensitizers (PSs) are one of the crucial elements of PDT. The therapeutic efficiency of these substances arises from their intrinsic features. PSs, by absorbing specific wavelengths of light, trigger photochemical and photophysical reactions [64–66]. A perfect PS must be chemically pure and have uniform composition to produce efficient ROS and selectively accumulate in the target tissue. In the absence of radiation, PSs are harmless. Absorption of light by PSs should ideally be in the phototherapeutic window which covers the long-wave part of the electromagnetic spectrum (range between 600 and 850 nm). PSs tend to be stable in solution, serum, or plasma and be easily deleted from the organism while being a cost-effective alternative to current treatment modalities [67–70].

The chemical structures of PSs are diverse and divided into three groups. The porfimer sodium and the HpD are known as the first-generation PSs. For alleviating the first-generation disadvantages such as light absorption at a particular spectral region, second-generation PSs had arisen. The second-generation PSs included the derivatives of chlorins, bacteriochlorins, and phthalocyanines, which show greater effect on the tumor site because of their deep-red region absorbance. Therefore, they increase light penetration. Finally, third-generation PSs by conjunction with target molecules or encapsulation into carriers are being developed to selectively target tissue regions. The progress in PS development is mainly aimed to improve PDT specificity and efficiency [64, 71, 72].

Riboflavin, berberine (Alkaloids), curcumin, anthraquinone, psoralen (furanocumarins), cercosporin, bergapten (furanocumarins), and thiophene are the most common photoactive natural PSs that are due to the use of medicinal properties in PDT [73].

The PS alone or in combination with several materials such as hydrogels, polymers, nanotubes, or organic metal frames (MOFs) can maintain the effectiveness of microbial inactivation and repair/regeneration processes [74]. PSs can be injected [74], rubbed [75], or sprayed on the wound site [76].

### Light

Different light sources have been used in PDT, such as lasers, incandescent light, and laser-emitting diodes (LED). Laser light sources are costly and demand an optical system to develop the light beam for irradiation of a larger tissue area. Non-laser light sources such as conventional lamps can be coupled to optical fibers to set the light wavelength. Nevertheless, conventional lamps cause thermal effects, which must be strictly avoided in PDT. Finally, LEDs have been introduced as a promising light source in PDT. LEDs have some advantages including affordable and less hazardous. They tend to produce less heat which causes non-destructive thermally action and are widely accessible in flexible arrays [77].

Light penetration acting very complex within the tissue. They can be reflected, scattered, or absorbed. According to the tissue type and the excitation wavelength of light, these processes are different. There is a competition in light absorption between endogenous chromophores existing in tissues like hemoglobin, myoglobin, and cytochromes and PS, which can cause reduction in the PDT process [78, 79].

Among the broad spectrum of light, ultraviolet (UV) light in the range of 100–400 nm can impair biological components. Therefore, its biomedical uses are limited, while visible light (400–650 nm) can be applied for various PS activation [80]. Additionally, “biological transparent windows” are divided into two windows: near-infrared (NIR)-I with a range of 750–1000 nm and NIR-II window with a range of 1000–1700 nm. In both windows, tissue absorption is low and there is ultra-low scattering, low autofluorescence, and maximum tissue penetration depth. Consequently, they can be used for biophotonic imaging [73, 74]. Sometimes, the tissue penetration depth of light can be restricted which can influence the number of activated PS. As the result, it affects the amount of ROS and generated singlet oxygen to kill tumor cells [74]. *λ* <650 nm usually has a lower penetration depth in tissues, while *λ* >850 nm ranges are not adequate to excite or activate PSs [75]. “Phototherapeutic window” is defined as the most appropriate wavelength for PDT with a 650–850-nm range.

### Oxygen

The third crucial element of PDT is oxygen, which is essential for ROS generation in PDT mechanism. The tissue oxygen levels directly affect PDT treatment efficiency, while oxygen levels are widely related to tissue density, and particularly in the deeper part, act as a limiting factor. Irradiation with a high light fluency rate can temporarily cause depletion of local oxygen and interrupt ROS generation which consequently reduce treatment effectiveness. Oxygen depletion results when the oxygen consumption level during PDT processes is higher than the level of oxygen diffusion in the irradiated area [76].

## Photodynamic therapy applications

PDT received increasing attention as a new treatment that is used for both malignant and non-malignant diseases, because of its non-invasive feature. PDT has many applications in a wide range of medical fields of oncology, dermatology, urology, ophthalmology, and dentistry and has shown efficient treatment in healing a broad range of diseases [81–85]. Due to the invasive nature of the regular treatment of cancers like radiotherapy, chemotherapy, surgery, and development in PDT appears to be a promising alternative in the localized and non-invasive treatment [67, 86, 87]. The main strength of PDT is selectivity treatment of tumor tissues while minimizing damage in non-malignant cells [85, 87].

### Cancer

The antitumor mechanisms induced by PDT include the following: the production of ROS leads to direct cellular damage, and indirect killing of tumor cells by knocking down the tumor vascular and causing patient’s immunostimulation by boosting cancer cell- derived antigen-presenting T-cells [88, 89].

### Infection

Antimicrobial PDT also known as photodynamic inactivation (PDI) is an efficient, safe, and affordable method to treat different infectious diseases [90]. Since the skin and soft tissue lesions are susceptible to infection by multidrug-resistant pathogens and cause a delay to heal, the role of PDT is more important and suggested as a solution. Furthermore, usual local treatments for infected wounds like burns, trauma, surgery, or diseases are costly and commonly ineffective [91, 92]. PDT provided excellent results in wound healing, promoting tissue repair by killing bacterial cells and stimulation of fibroblast proliferation [72]. Application of PDT in dental infections is growing as one major goal of modern therapy. According to recent studies, pathogens prevalent in the subgingival periodontal plaques such as *Staphylococcus* spp., *Streptococcus* spp., *Porphyromonas gingivalis, Fusobacterium nucleatuma*, and *Porphyromonas gingivalis* have been successfully destroyed by PDT, both in aqueous suspension and as a biofilm [93]. Further purpose of PDT is for managing mycosis. These infections are increasingly spread around the world, mainly because only three major types of antifungal drugs are available for invasive infections, and the efficiency of the treatment depends on the patient’s immune response [94]. PDT has shown good efficacy in the treatment of fungi disease through a proper formulation, including PS and keratolytic agents [95].

PDT has been revealed to be effective in the inactivation of mammalian viruses like hepatitis A, B, and C viruses, human papillomavirus (HPV), human immunodeficiency virus (HIV), herpes viruses, human parvovirus B19, human cytomegalovirus, adenoviruses, and enteroviruses [96–98]. PDT is widely used in pandemic research as an alternative or complementary therapy approach to target SARS-CoV-2 [99, 100].

### Treatment of vitiligo

Since oxidative stress is one of the causative factors in the pathology of vitiligo, it has been hypothesized that PDT can be effective in the treatment of this disease. The hypothesis has been examined in several studies. Rahimi et al. [101] treated vitiligo patches with topical 5-aminolaevulinic acid (5-ALA) and irradiated them with red light at 120 J/cm^2^ dose. The results showed no additional therapeutic impact of PDT in comparison with topical corticosteroids. In a similar study, Fernandez-Guarino et al. [102] observed no significant repigmentation on the facial vitiligo lesions of the patients after treatment with PDT. In another study, Giorgio et al. [103] compared the efficacy of PDT and micro needling in the repigmentation of vitiligo lesions but found no significant differences in treatment outcomes of these two methods. On the other hand, in a number of studies, the effectiveness of PDT in the treatment of vitiligo has been demonstrated. Zhang et al. treated vitiligo lesions with 1.5% 5-ALA, followed by 80 mw/cm^2^ red light irradiation and found that 5-ALA-PDT effectively repigments vitiligo patches to some extent [104]. Similarly, Serrano and colleagues reported some degree of repigmentation in vitiligo lesions treated with PDT [105]. Overall, although oxidative stress is one of the factors involved in the development and progression of vitiligo, and PDT is effective in modulating this factor, the results of these studies suggest that PDT alone could not be considered as an effective monotherapy method to reverse all the changes induced by complex causative factors of vitiligo.

### Treatment of psoriasis

PDT stimulates fibroblasts to secrete MMP-1 and 3. It also upregulates IL-10 expression while suppresses expression of transforming growth factor-beta (TGF-β) in cultured fibroblasts. Additionally, the secretion of IL-1β, IL-2, and TNF-α is increased in several immune cells treated with PDT [106–108]. This evidence suggests that PDT can be used as an effective treatment for inflammatory skin diseases. However, there are few reports on the effectiveness of PDT alone or in combination with other methods for treatment of psoriasis. In a study performed by Calzavara-Pinton and colleagues [10], the efficacy of PDT in treatment of psoriasis was observed only in 35% of patients. Moreover, combination of topical 5-ALA and PDT for treating chronic plaque psoriasis is not an efficacious method due to variable outcomes and severe pain after treatment. In addition, PDT combined with intense pulsed light used to treat nail psoriasis provides moderate effectiveness [109, 110]. However, early clinical studies of other modalities such as topical methylene blue and hypericin, as well as systemic 5-ALA and verteporfin, have demonstrated that these PSs are potent and much better tolerated than topical 5-ALA. The major limiting factor revealed in many of the studies was the side effect of pain and burning sensations related to PDT [111].

## Application of photodynamic therapy via ROS generation in dermatology

### Skin regeneration

ROS exist in a delicate homeostasis that is regulated by their host’s antioxidant capacity, and they play a key role in wound healing and adhesion formation [47, 112, 113]. Although ROS formation has previously been observed mostly within the first 2 h following cell injury, their impact on cell migration and proliferation can be detected for up to 24 h [114]. In vitro, manipulating cellular ROS has been found to slow fibroblast wound migration [115, 116] and to prevent the formation of postoperative adhesions in surgical animal models [112].

The importance of homeostatic levels of ROS and redox signaling in skin regeneration is well understood [47, 117, 118]. Physiological levels of ROS are required for vasoconstriction and thrombus development, which limit local blood flow. Early-onset ROS peak levels are linked to first platelet aggregation, which stimulates chemotaxis and adhesion molecule expression and allows platelets and inflammatory cells migrate to the site [119]. Second, the generation of ROS within tissue induces adherent leukocyte diapedesis across the vascular wall, resulting in microorganism death at the wound site. Neutrophils and macrophages produce high levels superoxide and H_2_O_2_ because of NADPH oxidase [119]. This oxidative burst, which is followed by a temporary downregulation of several ROS-scavenging enzymes, is the fundamental mechanism of bacterial death and wound infection prevention [120]. ROS also provide further signals that promote wound healing, as indicated by their ability to stimulate the release of TNF and platelet-derived growth factor (PDGF). Monocytes and macrophages, among other immune-competent cells, move to the wound site to help in decreasing pathogens [119, 120].

The proliferation phase requires redox signaling as well. TGF-β1 signaling, which results in migration, collagen and fibronectin synthesis, and basic fibroblast growth factor (FGF) expression, is mediated by ROS, which promotes fibroblast proliferation and migration and mediates TGF-β1 signaling [119]. Through vascular endothelial growth factor (VEGF) expression, ROS also stimulates angiogenesis, endothelial cell division, and migration for blood vessel reformation. ROS facilitated wound healing by stimulating fibroblast proliferation and migration, resulting in the development of ECM, keratinocyte growth and migration, and re-epithelialization [119]. ROS are also known to induce TGFα in fibroblasts [121]. The presence of ROS causes the latent TGF-complex binds to its receptor and triggers signaling pathways such SMAD2/3, PI3K, and JNK [122]. As a result, the transcriptional activity of profibrotic genes such *NOX4*, *SMA*, and *COL I* rises. Increased *NOX4* expression also leads to increase in ROS production [123], which activates additional ROS-dependent signaling pathways such NFB and JNK [124, 125]. Increased ROS can potentially induce irreversible DNA damage by oxidizing its bases. Together, increased ROS and activated TGF-signaling promote fibroblast cell proliferation and transdifferentiation into myofibroblasts, as well as excessive ECM deposition and fibrosis [126]. Keratinocyte growth factor (KGF) is another important component in epidermal regeneration [127]. ROS are capable of triggering KGF receptor activation and its internalization [128].

During photochemical reactions, the PDT dose is a significant element in determining ROS concentration [129]. Low-dose PDT promotes proliferation and differentiation without dramatically increasing cell death, in contrast to the cellular toxicities generated by high amounts of ROS [130]. As a result, pluripotent stem cells such as mesenchymal stem cells [131], osteoblast precursor cells [129], neural stem cells [132], and others are encouraged to differentiate. Exogenous ROS has been shown to have regulatory effects on stem cells in vitro [132]. In situ ROS generation in mouse skin recently increased hair follicle stem cell proliferation, increasing hair growth in the quiescent phase and enhancing burn healing [9]. PDT triggers a cascade of signals that can produce ROS such as HIF-1 and other cytokines such as TNF, VEGF, and interleukin (IL) such as IL-1 and IL-6, which in turn controlled the induction of several MMPs [133, 134]. MMP3 is a critical player in the disruption of collagen fibrils and the reorganization of cutaneous connective tissue following damage. MMP3 levels increased significantly after PDT, promoting keratinocyte and fibroblast migration, and possibly reflecting greater availability of growth hormones such as insulin-like growth factor (IGF) and other growth factors that can regulate neutrophils [135].

Previous studies have shown that acute inflammation can be stimulated by PDT, which results in wide changes in the physiological processes in infected, or non-infected chronic wounds and could enhance the healing mechanism [133]. PDT acts at various healing stages and overall enhances the tissue healing process when low doses of both PS and energy density have been applied. PDT improves skin texture and tone and reduces fine wrinkles through dermal remodeling. It also improves UV-induced lesions. Photodynamic rejuvenation does not cause scarring and its adverse effects are mild to moderate. It can be considered as a promising approach for skin rejuvenation with excellent short-term results and well tolerability [109].

PDT application can trigger cell proliferation in skin tissue and in vitro immortalized keratinocytes via ROS generation [136]. Carrasco et al. have been shown that after PDT utilization on various skin murine models (ulcers, severe thermal burns, scarring alopecia), there was a predictable increase of ROS that triggered cell proliferation at the bulge region of the pilosebaceous follicle, which is a significant stem cell replenisher. This stimulates hair growth, tissue repair, and wound healing [9]. Further analyses are essential to determine the relevance of these outcomes on human.

### Skin wound healing

A wound is the result of damage to an epithelial surface and its underlying connective tissues, which can be worsened by underlying tissue injury, disease, and poor tissue perfusion and oxygenation. Acute wounds heal normally after surgery, burns, or trauma within 30 days due to optimal hemostatic and inflammatory cascades with tissue repair and regeneration, while chronic wounds do not heal within a normal time frame due to a disruption of these phases and persistent underlying pathologies, especially infection [137, 138].

Numerous growth factors, a well-organized ECM, and responsive cell populations characterize the microenvironment in a normal wound bed. Matrix synthesis exceeds matrix degradation, and the presence of MMP inhibitors (TIMPs) controls MMP activity. Normal wound angiogenesis and neovascularization occur in a timely manner, according to well-controlled sprouting of existing blood vessels and recruitment of endothelial progenitor cells (EPC). Finally, unlike chronic wounds, acute wounds are typically associated with a low bacterial burden. Bacterial biofilms are common in chronic wounds, causing chronic inflammation, excessive proteolysis, and destruction of essential growth factors, receptors, and/or ECM. Because there are neither functional receptor nor suitable promigratory matrix substrates, cells in these wounds are unable to proliferate and/or migrate efficiently. Insufficient oxygen and nutrition delivery for the cells dwelling within the wound bed are both features of chronic wounds, which lead to increased wound bed mutilation and impaired healing [139, 140].

D.D. Hartmann noted photobiomodulation (PBM) as a common intervention for skin damage, to characterize its impact on various stages of wound healing. PBM was able to modulate the inflammatory phase, especially on the first day. In the inflammatory phases, while PBM causes alternation in the cell redox potential, ROS level has been increased [141]. This shift in redox state mediated signaling pathways that activated nucleic acid synthesis, enzyme activity, protein synthesis, and cell cycle progression. ROS levels followed by oxidative pathways play important role on mitochondrial function in the inflammatory stages [142]. Results indicated that PBM can be considered as an effective treatment in the tissue repair process via increasing ROS level and subsequent signaling pathway. Also, PBM can be considered as a potential tool for manipulating exosome secretion as they have been used recently in much research for wound healing and tissue regeneration [143].

As a matter of fact, low-dose PDT could play a role same as PBM therapy, with the variance that in low-dose PDT, there is a specific targeting to cells that have received the PS. In PBM, there is no specific targeting to cells in the wound area, and all cell types will be similarly revealed to light. But in low-dose PDT, the PSs will be taken up into specific cells. Other studies have demonstrated that in the normal wound healing process, low levels of ROS generated for a brief time interval can mediate intracellular signaling for collagen deposition and cell proliferation [144]. Low levels of ROS and high levels of antioxidants are essential for normal tissue repair [145]. In our recent research, it has been seen that at low concentration of 5-ALA (5 μg/mL) and low irradiation energy density of 1 J/cm^2^ (low-dose PDT) in both normal and diabetic cell models caused a slight increase in ROS levels compared to control groups which lead to better wound closure in those group. This could note the good impact of low-dose PDT on wound healing and affirms prior observations [11]. PDT contributes in different forms to the wound healing procedure: causing bacteria death, decreasing or raising inflammation, promoting fibroblast proliferation. Consequently, collagen and elastin formation increase TGF-β and MMPs. According to this, PDT provided good consequences in the wound healing process, acting in different phases of tissue repair [146]. There is evidence of a strong cellular infiltrate response in the treated chronic wound after PDT. Recently, it has been shown that after PDT in chronic wounds, there is a significant enhancement in certain inflammatory cells, such as TNF alfa+ mast cells (MCs), T regs, plasmacytoid dendritic cells (DCs), MHCII positive dermal DCs [147], and macrophages [133], and TGF-β which is directly related to increasing wound repair. TGF-β seems to act in early phases of wound healing, where it possibly induces an epithelial–mesenchymal transition, allowing the keratinocyte migration from the borders toward the wound’s bed [148]. TGF-β is also able to promote the myofibroblast differentiation which has an important role in wound healing. In some studies, an increase in fibroblasts has been seen after PDT of chronic wounds compared to the control group. Additionally, MCs may send signals for the recruitment and differentiation of fibroblasts which are associated in the chronic wound healing. It has been noted that, after PDT, the number of MCs increase and undergo degranulation [149]. Upon PDT, MCs are not only recruited, but also must be activated to secrete in response to treatment. The papillary dermis vessels seem to be the main site of cells after PDT and it can be suggested that endothelial cells can regulate the recruitment of MCs at this location [150]. The current literatures indicate that PBM can be a potent short-term way to decrease oxidative stress markers (e.g., thiobarbituric acid reactive) and to enhance antioxidant contents (e.g., CAT, GPx, and SOD). It seems low-dose PDT can act as PBM in this regard for wound healing [151].

The local oxidative stress created by PDT is antagonized in cells by three primary antioxidant mechanisms: SOD, CAT, and the GSH system [152]. Small antioxidant molecules such as vitamin E and ascorbic acid supplement the protective activity of antioxidant enzymes against ROS [153]. Low ascorbate concentrations (e.g., 0.5 mm) raise PDT effectiveness, but at higher concentrations (e.g., 10 mm), it has an antioxidant effect [154]. Exogenous oxygen radicals induced by PDT can directly peroxidase polyunsaturated fatty acids (PUFAs) and create lipid autoxidation, coinciding with cellular GSH depletion [155]. Numerous examinations have shown that GSH removal increases the induced toxicity of PDT [156], which is attributed to GPX-based detoxification [157]. Intracellular utilization of glutathione has been shown to be a predictor of PDT efficacy in that cell. Cells that express higher levels of GPX enzymes are resistant to PDT, and cells that express higher levels of GSTP1 can detoxify xenobiotic compounds and photosensitizers [158]. The potentiation of PDT with inhibition of antioxidant enzymes has been less examined. However, inhibition of SOD enzymes with 2-metoxyestradiol (2-ME) [159], inhibition of CAT by 3-amino-1,2,4-triazole (3AT), depletion of intracellular glutathione with buthionine sulfoximine (BSO) [160] or mercaptosuccinic acid, and combinations of antioxidant inhibitors have been shown to potentiate the antitumor effect of PDT [161, 162].

**Fig. 2:**
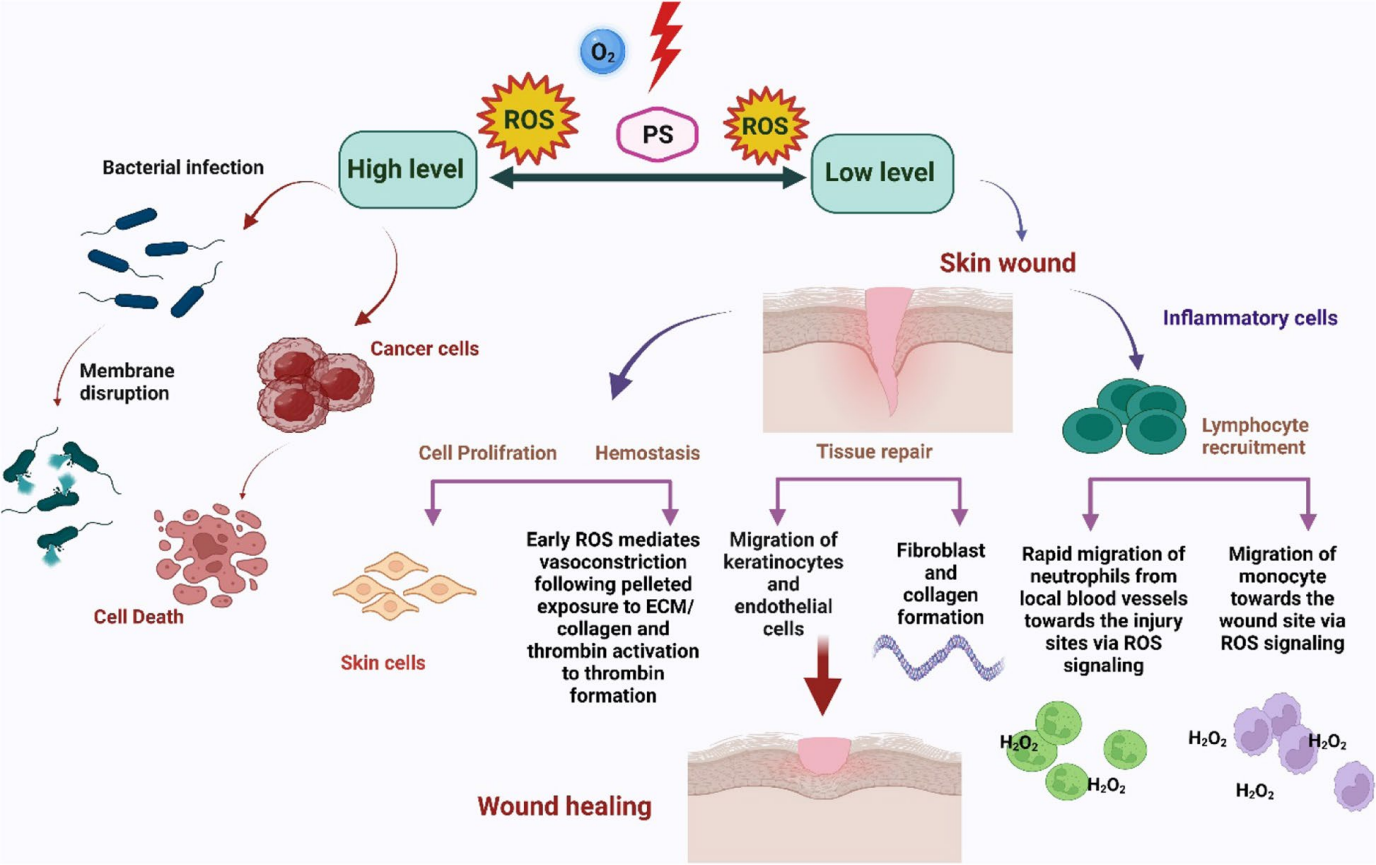
ROS produced from the PDT process according to its intensity can be used for cancer or infection treatment or applied for wound healing

As it has been shown in Fig. 2, after PDT process according to level of ROS production, there are two pathways. If the amount of ROS is at a high level, the PDT applies for cancer treatment (antitumor effect of PDT) and antibacterial purposes. However, when the produced ROS is in a low level, it can be applied for wound healing and cell proliferation approaches. This effect can be through migration of keratinocytes and endothelial cells, and also fibroblast and collagen formation. Another way is the effect on inflammatory cells such as rapid migration of neutrophils and monocytes from blood vessels toward the wound site (Fig. 2).

## ROS-responsive materials along with PDT for skin wound healing

Low-dose production of ROS is typically continuous and is necessary in the regulation of various biological phenomena. Therefore, the change of the oxidative stress level and condition in antioxidant/oxidant composition has been detected as one of the most important parameters of aging-related bio action and is one of the factors in the onset and development of degenerative / chronic diseases (e.g., cancer, neurodegenerative disease, atherosclerosis) [163].

Redox signals and increased oxidative stress act by facilitating and providing conditions for homeostasis, inflammation, granulation tissue formation and development, angiogenesis, wound closure and formation, completion, progression, and suppuration of the ECM. Hence, ROS play an essential role in regulating the natural healing of wounds in most kind of wounds. Therefore, to fight invading bacteria and microorganisms as well as guide cells to the wound site, producing small concentrations of ROS is necessary for repair [164].

However, excessive and uncontrolled oxidative stress leads to the maintenance of inflammatory conditions and failure to regulate these processes that plays a critical role in the pathogenesis of chronic non-healing wounds [165]. As mentioned above, a sensitive balance for positive effects of ROS and their injurious effects is very important for ulcer treatments and proper wound healing. So, as mentioned, although the generation of ROS is important and necessary to start wound healing, the production of excessive concentrations of reactive oxygen compounds is harmful for wound healing. Due to persistent oxidative stress with high concentrations of ROS, there is fat peroxidation, changes in proteins structure, and DNA damages, and it has been shown to impair wound healing by increasing apoptosis and cellular aging [166]. Clinical studies on chronic wounds show that non-healing wounds are in a state of severe oxidative stress, which leads to impaired wound healing. Some conditions such as hyperglycemia and tissue oxygen deficiency are often associated with high oxidative stress level [149]. In diabetic patients, standard methods of wound care usually involve debridement, antibiotic application, the use of wet/moisture wound dressings, and local pressure reduction on the wound. Recent research and development often focus on specific parameters of the diabetic wound environment, including growth factor topical treatment, the use and insertion of bone marrow-derived endothelial cells and epithelial cells, and tissue engineering-based collagen tissue transplants. As a different approach, precise control of the levels of ROS through antioxidants and antioxidant enzyme systems may reduce cell damage caused by oxidative stress [167].

Clinical studies have shown that diabetic wounds that do not heal are involved in a highly oxidative environment that is associated with hyperglycemia and tissue hypoxia, leading to delayed wound healing. People with long-term type 2 diabetes have a notable decrease in the antioxidant enzyme activity. Oxidative stress may affect the healing of diabetic ulcers through skin damage, neuropathy, ischemic lesion, and local infection [168].

In general, under normal conditions, endogenous ROS is sustained at low concentrations by intracellular redox equilibrium. However, when the redox equilibrium is unbalanced, ROS production increases that is related to cellular pathological conditions, including the initiation and development of inflammation. The concentration of ROS in pathological sites, such as activated immune cells or cancerous cells, can reach up to 100 × 10^−6^ M, two or three times more than in normal cells (≈20 ×10^−9^ M) [169].

By considering such heterogeneity of ROS concentration in the tissues, researchers work on design and synthesis of ROS-responsive materials to target inflammation, cancer cells, and wound sites to manage ROS in pathological regions. In the last decade, various substances that are sensitive to ROS compounds have been designed to use for increasing the concentration of ROS in pathological areas for cell growth inhibition or even direct cell death induction. Also, such materials can be designed for decreasing the level of ROS to a standard value to reduce overgeneration of oxidative stresses in tissue and then relieve inflammation. Also, ROS-responsive substances can be used for targeted imaging of inflammation tissues [103]. One of the major applications of ROS-responsive nanostructures is their applications in the ROS activated drug delivery systems. They can be used in the ROS-responsive gene delivery, upregulation of ROS in malignant cells, scavenging of ROS in inflamed cells, and ROS linked imaging and probes in detections. Using ROS-sensitive chemical compositions in the chains of polymers or copolymers can induce the ability to selective ROS controlled polymerization for drug delivery purpose. For instance, selenium and tellurium nano-compounds have recently attracted the attention of many researchers due to their excellent ROS sensitivity. Mono- and di-selenide-containing polymers are insoluble in aqueous solutions and are used in the fabrication of amphiphilic block copolymers. Telluride is less toxic and more sensitive to ROS than selenide and sulfide due to its lower electronegativity which make it a suitable composition in design and synthesis of copolymers and polymers for drug delivery systems and ROS-responsive prodrug preparation [104]. The prodrugs consisted of three domains, a ROS acceptor that can be sensitive to ROS, an effector that is the native drug part, and a linker between the ROS acceptor and the effector. Generally, they are chemical compounds which after administration can convert to the active drugs via chemical or enzymatic activation. Prodrugs are designed to increase the solubility, achieve targeted delivery, and facilitate cell internalization of drugs. Recently, ROS-activatable prodrugs have been designed and developed by caging the native drug with ROS-cleavable moieties. ROS-responsive prodrugs can be mainly divided into small-molecular, protein, and polymer prodrugs [104].

To prevent the production of excess ROS around the injury site, advanced biomaterials can be remodeled to release their cargos in an injury microenvironment to regulate the elevated levels of the ROS, which may also help to downregulate the oxidative stress and promote tissue regeneration. A variety of scaffolds and bioactive materials have been notified to help the regeneration of damaged tissues based on the scavenging of free radicals and reactive species that give high protection to the tissue function [170].

To reduce the ROS level in the wounds, several ROS-scavenging materials have been incorporated into hydrogel dressings, such as antioxidants, enzymes, and nanomaterials [171]. Hydrogels with injectable and antioxidant properties can provide sustained release and potential benefits for wound healing [172]. Interestingly, the ROS-scavenging hydrogels can also be modified by using a ROS-responsive linker. In the presence of ROS-sensitive linkers, such hydrogels were able to consume excessive ROS and induce drug release to inhibit bacterial infection, modulate inflammatory response, and promote angiogenesis and wound healing [173–175].

Bacterial-infected wounds such as diabetic foot could not heal quickly. Nanoparticles (NPs) exhibiting light-responsive multifunctional properties were designed as an enticing platform for the management of bacterial-infected wounds which can be use along with PDT. To leverage synergistic chemical and PDT for bacteria contaminated skin wounds, Wang et al. developed photosensitizer chlorin e6 (Ce6) and magnesium (Mg)-containing nanocomplexes [176, 177]. The multifunctional NPs could efficiently generate ROS under laser irradiation to kill the bacteria. Additionally, ROS-responsive release of Mg2^+^ from the NPs could induce cell proliferation and migration and significantly enhancing wound repair. Therefore, ROS-responsive biomaterials can be developed into various shapes and structures, such as nanoparticles, hydrogels, nanofibers, microneedles, and so on to enhance their application in wound healing and tissue regeneration.

## Technical advances in photodynamic therapy

As a non-invasive and rapidly developing method, PDT has been widely tested for tumor ablation and infection control. Recently, NP-based PDT has aroused significant attention from many researchers in improving wound healing. There are different nanoparticles for PDT applications [178]. In general, NPs can be divided into two categories: organic and inorganic. Organic NPs such as polymers, liposomes, and micelles are in the category of organic nanoparticles, and inorganic nanoparticles are compounds that do not contain carbon and are made of metals and metal oxide-based NPs [179]. Both organic and inorganic nanoparticles have been used for wound photodynamic treatment. In most cases, organic nanoparticles are more suitable candidates for PS in PDT due to their high biodegradability and biocompatibility [180]. The difference between these two types of NPs is based on morphology, spectral ranges, and heating efficiency. The typical NPs that can be utilized for PDT depends on the laser power to be operated and the site of wounds to be treated [181]. By combining laser light and light-absorbing NPs, NP-based PDT has recently emerged for wound healing treatment. By applying light-absorbing NPs, photodynamic heating occurs mainly in the NP-concentrated tissues, thereby increasing the temperature more in the targeted wounds compared with the normal tissue around the wound. By photodynamically tuning the temperature of the wounds, NP-based PDT may promote cell proliferation and generate ROS, thereby playing a beneficial role in tissue regeneration [182, 183].

NP-based PDT can be very effective in sealing skin wounds and inducing soft tissue regeneration. Skin wounds, especially open skin wounds, are often associated with bacterial infections that can cause serious complications and impede the wound healing [184–186]. NP-based PDT have a high potential for wound sterilization through the production of ROS mediated by oxidative damage to biomolecules (such as proteins, nucleic acids, and lipids) of pathogenic microorganisms [187, 188]. Different types of NP base PS with antibacterial properties have been used to reduce bacterial infections [189, 190]. Here we bring some examples of nanoparticles use in PDT. Carbon nanoparticles are available in three forms: fullerene, diamond, and graphite. The specific alignment of the carbon atoms in fullerene creates unique thermal properties. CNTs with a tubular structure are one of two forms of fullerenes and have been extensively studied for PDT purposes due to their chemical stability and high tensile strength [191]. Among these compounds, graphene-based nanoparticles are combined into poly(vinylidene) fluoride membranes by electrospinning to generate heat and regulate the local temperature under NIR light irradiation [192]. After 5 min of irradiation, the local temperature increases, ROS is produced, and NIR light irradiation leads to the death of gram-positive and gram-negative bacteria. GO / PVDF composite membrane improves wound healing by reducing wound infection [179]. Black phosphorus-based (BP-based) NPs have excellent biocompatibility and are broadly studied. The degradation derivatives (such as H_2_O, CO_2_) of BP are inoffensive and can conform as critical nutrient components for bone tissues. However, the instability of BP NPs in the atmosphere has limited the clinical use of these compounds. Huang et al. increased the stability of BP combined with SF, as an exfoliating agent, into BP nanosheets [193]. PDT treatment with NP-based BP has been shown to significantly improve skin wounds and prevent *E. coli* infections in mice skin wounds [193]. The skin wounds treated with BP-based scaffolds healed with an intact epidermis under irradiation of NIR laser light [182].

Lipid-based NPs and solid lipid NPs (SLNP) are capable to promote re-epithelialization in the restoring procedure. It was suggested it can decrease the pain and accelerate keratinocyte proliferation, differentiation, and migration. Studies of wounds in diabetic rats have shown that these lipid carriers can accelerate wound closure. SLNP and nanostructured lipid carriers (NLC) have no limitations of liposomes. SLNP and NLC-loaded growth factor was frequently utilized for chronic wound therapy [194].

Nanoemulsions are obtained by shearing a mixture of two immiscible liquid phases (oil and water) and one or more surfactants. Stable droplets are formed with diameters between 50 and 500 nm. SLNP and NLC packed growth factors were prepared through the emulsification-ultrasonication technique for the higher encapsulation efficiency in the wound therapy [195].

Polymeric materials have been used as the most adaptable and suitable compounds for nanocarrier systems. These polymeric nanoparticles are popular in both biomedical and bioengineering. Properties of these compounds may be adjusted by changing a variety of factors such as composition and sequence of the polymer units used, molecular weight, the degree of branching, confirmation of the chain, and crosslinking density. The unique properties of these polymers such as solubility, biocompatibility, hydrophilicity, and flexibility of the main chain could release the desired drugs in a specific site with high protection from the wound environment. Synthetic and natural polymers (such as dextran, PEG, and poly (vinyl pyrrolidone) (PVP), streptavidin, poly-lysine, PEI) have been employed on the surface of the NPs. Currently, NPs are based on polymer materials such as poly (lactidecoglycolide) (PLGA), polycaprolactone (PCL), PEG, alginate, gelatine, and chitosan, as well as in mixtures which have been employed as wound dressings in wound care applications [196]. Polymeric nanocarriers such as polymeric NPs [192], polymeric micelles [197], polymersomes [198], and dendrimers have been used in photodynamic treatment [199]. Among the polymeric NPs, polysaccharides are the most available nanomaterials employed in wound administration. Chitosan [200], dextran, alginate, and cellulose derivatives are broadly used in different wound care applications [201]. NPs in methylcellulose formulations, HemCon bandage, demonstrated antibacterial and anti-fungi activities, wound healing acceleration, and minimization of scar formation [202]. PLA and PGA homopolymers, biodegradable polymers such as PLGA for PS encapsulation, PLGA-curcumin NPs, PCL NPs compacted chitosan/enoxaparin with chitosan, biodegradable poly (b amino esters) and maleic acid, and PBAE NPs are used for wound healing applications [203, 204].

## Pros and cons of photodynamic therapy

A major, unique advantage of PDT is its non-invasive nature and quick action that could effectively rejuvenate photo-damaged skin, while successfully treating a range of dermatologic conditions, including prevention and therapy of pre-cancerous actinic keratosis. The alternative to PDT is usually surgery, which leaves a scar or application of anticancer creams, which although effective cause quite significant inflammation (redness and crusting) for several weeks. PDT usually leaves no visible scar. Depending on the type of skin lesion, your dermatologist may suggest surgery, cryotherapy, or other treatments beside PDT [205].

PDT also has drawbacks. It can only treat areas where light can reach. It means that it can only be used to treat some cancer on or just under the skin, or in the linings of some organs. Usually, the light used in PDT cannot pass through more than about 1/3 inch of tissue, or 1 cm. Photosensitivity is another common complication in PDT, which can last for months [206], although nowadays many approaches have been suggested for overcoming these disadvantages such as using fiber optics for more light penetration in depth tissue and application of novel PSs that induce less photosensitivity in patients. However, PDT application in the clinic should be done with caution.

## Conclusion

Significant improvements and understanding of the underlying mechanisms that drive PDT as a therapeutic modality have been made. A review of the literature clearly indicates that low-dose PDT holds an important role in the treatment of variety of diseases. In this paper, clear evidence from a few different studies suggest that PDT not only holds promise as an important tool to treat diseases where destruction or cell death is required but additionally it may become an important tool in addressing the skin regeneration. By implication, PDT may become useful in stimulating cellular processes involve in regenerative medicine such as wound healing. Considering the importance of ROS and the role it plays in different molecular and cellular processes as well as the activation of signaling pathways, using PDT as a mechanism to induce the formation of ROS is evident. The distinguishing factor clearly lies in the application and dosimetry. This remains a highly contentious issue as many contradictory results have been published where dose and light parameter selection influence the outcomes. Under normal, disease-free conditions, biological systems maintain a fine balance in the production, use, and termination of ROS. However, under diseased conditions, this balance is disturbed and using therapeutic modalities to manage ROS production may then aid in better wound healing and tissue regeneration.

## Data Availability

The data presented in this study are available in this manuscript.
